# The *BrGI* Circadian Clock Gene Is Involved in the Regulation of Glucosinolates in Chinese Cabbage

**DOI:** 10.3390/genes12111664

**Published:** 2021-10-22

**Authors:** Nan Sun Kim, Su Jeong Kim, Jung Su Jo, Jun Gu Lee, Soo In Lee, Dong Hwan Kim, Jin A. Kim

**Affiliations:** 1 Department of Agricultural Biotechnology, National Institute of Agricultural Science, Rural Development Administration, Jeonju 54874, Korea; nskims@korea.kr (N.S.K.); silee@korea.kr (S.I.L.); 2 Department of Plant Science and Technology, Chung-Ang University, Anseong 17546, Korea; tnwjd9701@naver.com; 3 Department of Horticulture, College of Agriculture & Life Sciences, Jeonbuk National University, Jeonju 54896, Korea; jjs446@naver.com (J.S.J.); jungu@jbnu.ac.kr (J.G.L.)

**Keywords:** Chinese cabbage, circadian clock, glucosinolate, metabolite, RNAi

## Abstract

Circadian clocks integrate environmental cues with endogenous signals to coordinate physiological outputs. Clock genes in plants are involved in many physiological and developmental processes, such as photosynthesis, stomata opening, stem elongation, light signaling, and floral induction. Many *Brassicaceae* family plants, including Chinese cabbage (*Brassica rapa* ssp. *pekinensis*), produce a unique glucosinolate (GSL) secondary metabolite, which enhances plant protection, facilitates the design of functional foods, and has potential medical applications (e.g., as antidiabetic and anticancer agents). The levels of GSLs change diurnally, suggesting a connection to the circadian clock system. We investigated whether circadian clock genes affect the biosynthesis of GSLs in *Brassica rapa* using RNAi-mediated suppressed transgenic *Brassica rapa* *GIGENTEA* homolog (*BrGI* knockdown; hereafter GK1) Chinese cabbage. *GIGANTEA* plays an important role in the plant circadian clock system and is related to various developmental and metabolic processes. Using a validated GK1 transgenic line, we performed RNA sequencing and high-performance liquid chromatography analyses. The transcript levels of many GSL pathway genes were significantly altered in GK1 transgenic plants. In addition, GSL contents were substantially reduced in GK1 transgenic plants. We report that the *BrGI* circadian clock gene is required for the biosynthesis of GSLs in Chinese cabbage plants.

## 1. Introduction

Organisms are exposed to daily environmental cycles of light and temperature. These 24 h cycles trigger an endogenous biochemical oscillator known as the circadian clock in almost all living organisms. This circadian clock coordinates internally generated rhythmic behaviors and biological processes and functions to anticipate the environmental changes associated with the day–night cycle [1]. Synchronization of endogenous physiology and metabolism with the rhythmic environmental cycles is crucial for plant fitness and adaptation to environmental challenges [2,3]. The circadian clock plays an important role, especially in fossil plants, in regulating numerous physiological processes and internal metabolic and hormonal signals [4,5]. One-third of the genes of the model plant *Arabidopsis thaliana* oscillate cyclically as a function of light and/or temperature [6,7], regulating a variety of processes such as hypocotyl and root growth [8], flowering time [2], sugar metabolism [2], photosynthesis [9], nutrient homeostasis [10], and hormonal signaling [11]. The circadian clock is composed of interlocked feedback loops regulated both transcriptionally and post-transcriptionally, including by post-translational modification and protein turnover, which drive the rhythmic behavior of genes, proteins, and metabolites [6,12,13,14,15].

Multiple genes involved in secondary metabolite biosynthesis have diurnal expression patterns [16], and secondary metabolism alterations can influence the circadian clock [17]. Based on clock gene mutant studies, clock genes regulate growth, photosynthesis, and metabolite synthesis [4,5] and improve productivity and metabolite synthesis in crop plants [18,19,20,21]. The circadian clock gene *GIGANTEA* (*GI*) is reported to have many important pleotropic functions such as flowering time regulation, light signaling, hypocotyl elongation, control of the circadian rhythm, sucrose signaling, starch accumulation, chlorophyll accumulation, transpiration, herbicide tolerance, cold tolerance, drought tolerance, and miRNA processing [22]. Since *GI* mutants were first described as late flowering mutants, several *GI* mutants with distinct phenotypes, obtained by random mutagenesis or T-DNA insertion, have been described in Arabidopsis [23,24,25]. Because *GI* null mutants are not lethal, despite its pivotal roles, *GI* mutation can improve crop traits. *GI* functions in abiotic stress tolerance, and loss of *GI* function enhances salt, cold, and drought tolerance in plants [18,26,27,28,29]. Therefore, *GI* mutation in crops can improve yield.

Glucosinolates (GSLs) are plant secondary metabolites that are derivatives of sugars and amino acids. GLSs play a major role in plant defense and affect the palatability and nutritional value of edible crops [3]. Some GSLs and their degradation products have anti-carcinogenic and antioxidant activities in humans and impart tastes and flavors to *Brassica* vegetables [30]. GSL biosynthetic pathways and products are regulated by multiple genes, as well as by light quality, abiotic stress, and temperature [31]. GSLs are a major class of sulfur-containing secondary metabolites involved in plant defense against pathogens. Many regulatory links between GSL biosynthesis and sulphate assimilation have been established. Sulphate assimilation has a diurnal rhythm and is light-regulated [32]. GSL biosynthesis is diurnally controlled by the light–dark cycle. The levels of GSLs and glutathione were higher during the day than night [33]. Additionally, genetic perturbations in the GSL pathway can influence the transcript abundance of core oscillator genes [6], while GSL genotypes altered the periodicity of a clock output unrelated to GSLs, i.e., the photochemical state of photosystem II. This suggests that the GSL pathway in plants is not simply related to carbon/nitrogen/sulfur flux, but also to growth-defense tradeoffs via circadian clock regulation [17].

The *B. rapa* Chinese cabbage is a plant belonging to the polyploid genus Brassica, and along with bok choy, turnip, and broccolito, it is an important agricultural crop worldwide. After multiple genome duplications and diplidization, circadian clock genes were preferentially retained relative to their neighboring genes in the *B. rapa* genome [34,35]. In previous studies, candidate paralogs of circadian clock-related genes were identified, and their diurnal expression pattern was assessed [36]. Additionally, RNAi-mediated suppression of GI expression in *B. rapa* increased tolerance to salt stress [18].

Here, we focused on GSL metabolism in a knockdown transgenic *B. rapa* line of *GI*. We investigated the correlation between *GI* and biosynthesis of GSLs using transcriptomic tools and metabolic analysis. There was a relationship between changes in clock function and changes in GSL metabolites. Our results suggest that manipulating the clock gene expression could improve the productivity and functionality of crops.

## 2. Materials and Methods

### 2.1. Plant Materials and Growth Conditions

The inbred line DH03 (*Bassica rapa* ssp. Pekinensis) and “*GIGANTEA* reduced GK1” [18] are used in this study. Seeds were soaked in distilled water for 8 h, sowed in a plastic pot containing vermiculite soil, and watered with tap water. Each tray was transferred to a growth chamber (Hanbaek Scientific Co., Busan, Korea) in the Department of Agricultural Biotechnology, National Academy of Agricultural Science, RDA, South Korea. Seedlings were grown in a controlled environment growth chamber under a 16/8 h light/dark cycle at 23 °C with cool-white fluorescent illumination (100 mol m^−2^ s^−1^, FLR40D/A fluorescent tube; Osram, Korea). After 8 days, the fresh weight, root length, hypocotyl length, and cotyledon area of 20 sprouts were measured and the other sprouts were harvested using liquid nitrogen. The frozen samples were lyophilized and ground to fine powders for further analysis. For each experiment, three replicates were used for analysis.

### 2.2. Soluble Sugar Assay

Freeze-dried powder (0.2 g) was extracted with 6 mL of 80% ethanol. The mixture was incubated at 65 °C in a water bath for 20 min and the supernatant was collected. The precipitate was extracted by the same process two times. The supernatants were combined and concentrated, and soluble sugars were analyzed by a high-performance liquid chromatography (HPLC) system using a Waters 600 separation module and Waters 2414 RI detector (Waters Corp., Milford, MA, USA). Sample (20 µL) was injected onto a Sugar-Pak I column (Waters, 300 mm × 6.5 mm) with water containing 0.0001 M calcium/EDTA. The sugar content was quantified according to the external standard method. For each sample, there were three biological repeats. The sugar concentration is expressed as mg/kg dry weight (DW).

### 2.3. Antioxidant and Antioxidant Capacity Assays

#### 2.3.1. Total Phenolic Content Assay

Total phenolic content was analyzed by the Folin–Ciocalteu colorimetric method, as described previously [37] with some modifications. Powder (200 mg) was mixed with 5 mL of 80% methanol and extracted in a water bath shaker set at 50 °C and 120 rpm for 60 min, followed by centrifugation at 4000× *g* for 15 min at 4 °C. Briefly, 100 μL of extract was mixed with 500 μL of water, to which 100 μL of Folin–Ciocalteu reagent was added. Next, 1000 μL of Na_2_CO_3_ (7%) was added, and the mixture was vortexed and kept in dark conditions. After 1 h, the absorbance at 760 nm was measured using a Biomate5 Spectrometer (Thermo Fisher Scientific, Waltham, MA, USA). Gallic acid standards were used to generate a calibration curve. Results are expressed as milligrams of gallic acid equivalent (mg GAE·g^−1^DW).

#### 2.3.2. Total Flavonoids

Total flavonoid content was analyzed using a colorimetric method, as described previously [38] with some modifications. The extract used for TPC analysis was also used for flavonoid analysis. Briefly, 200 μL of extract was mixed with 1000 μL of water, followed by the addition of 50 μL NaNO_2_ (5%). After 5 min, 150 μL of AlCl_3_·6H_2_O (10%) and 500 μL of NaOH (1 M) were added, and the absorbance at 510 nm was measured using a Biomate5 Spectrometer (Thermo Fisher Scientific). Catechin hydrate solutions of various concentrations were used as standards. Results are expressed as milligrams of catechin hydrate equivalent per gram on a DW basis (mg CE·g^−1^DW).

#### 2.3.3. Total Antioxidant Capacity Assay

The 2,2-diphenyl-1-picrylhydrazyl (DPPH) assay is based on a change in color (from violet to yellow) of DPPH. First, a mixture of 50 ppm DPPH solution in 100% methanol was prepared. Aliquots of the reaction mixture, consisting of 250 μL sample extract, or ascorbic acid as a negative control in methanol, and 3.75 mL of DPPH dissolved in methanol, were vortexed for 10 s and placed in the dark for 30 min. The absorbance at 517 nm was measured using a Biomate5 Spectrometer (Thermo Fisher Scientific) and free radical-scavenging activity (%) was calculated as described previously [39].

### 2.4. Extraction of Desulfo-Glucosinolates and Ultra-Performance Liquid Chromatography

Desulfo-glucosinolates (DS-GSLs) were obtained using previously reported procedures [40,41]. Briefly, crude GSLs were extracted from 100 mg of freeze-dried powdered seedlings with 2 mL of methanol (70%, *v*/*v*) by warming at 80 °C for 60 min in a water bath. After centrifugation at 2000 rpm for 10 min at 10 °C, the supernatants were collected. The extracts were loaded in a mini-column previously packed with DEAE resin, which was sealed and left overnight at room temperature after adding 200 μL of sulfatase. The column was washed three times with 0.5 mL of thrice-distilled water, and the eluted product was filtered through a 0.2 μm syringe filter before analysis. Sephadex A-25 was added to the appropriate reagent bottle, washed, and precipitated twice with distilled water; after 10 min, the distilled water was decanted and the remaining slurry was mixed with 100 mL of 0.1 M sodium acetate (pH 4.0). After 24 h, ultra-performance liquid chromatography (UPLC) was performed using an ACQUITY H-Class System (Waters).

### 2.5. Library Preparation and RNA Sequencing

Total RNA from *B. rapa* sprouts of DH03 and GK1 was isolated using the RNeasy Plant Mini Kit (Qiagen, Valencia, CA, USA), following the manufacturer’s instructions. The quantity and quality of RNA were checked with a 2100 Bioanalyzer (Agilent, Santa Clara, CA, USA); only samples with an RNA integrity number > 8 were used for library preparation. Preparation of each single-end complementary DNA (cDNA) library was conducted according to the TruSeq RNA Sample Preparation Guide (Illumina, San Diego, CA, USA). The cDNA libraries were sequenced using an Illumina HiSeq2000 Sequencer. Base calls were made using CASAVA software (Illumina, San Diego, CA, USA). Library preparation and RNA sequencing were performed in eGnome Inc. of Seoul, South Korea.

### 2.6. Transcript Quantification, Differential Expression Analysis, and Gene Annotation

Pair-end reads were cleaned by processing with PRINSEQ-lite v. 0.20.4 software (http://prinseq.sourceforge.net/, accessed on 20 August 2021). Sequences < 50 bp long, with at least one quality score < 10, a mean quality score of <20 (exact duplicates or reverse-complement exact duplicates were filtered out), and a quality score threshold of 20 were trimmed from both the 5′ and 3′ ends [42]. The clean reads of each sample were aligned to the reference transcriptome (*B. rapa*_197_transcript_primaryTranscriptOnly.fa of Phytozome V9.0, https://genome.jgi.doe.gov/portal/pages/dynamicOrganismDownload.jsforganism=Phytozome, accessed on 20 August 2021) using Bowtie software [43]. RSEM v. 1.3.0 software (http://deweylab.github.io/RSEM/, accessed on 20 August 2021) was used to generate read counts and the trimmed mean of M-values (TMM)-normalized fragments per kb of exon per million (FPKM) reads mapped for each transcript [44]. For differential expression analysis, negative binomial dispersion across samples was calculated using EdgeR v. 3.16.5 software [45]. Genes with more than a twofold change in expression, and a false discovery rate (FDR)-adjusted *p*-value 0.05, were considered differentially expressed. Information on gene annotation was supported by the *B. rapa*_197_annotation profile of Phytozome v. 9.0 (https://phytozome.jgi.doe.gov, accessed on 20 August 2021). Gene Ontology (GO) enrichment analysis of RNA sequencing (RNA-seq) data was performed using ShinyGO v. 0.66 (http://bioinformatics.sdstate.edu/go/, accessed on 20 August 2021).

### 2.7. Quantitative Real-Time PCR Expression Analysis in B. rapa

Quantitative real-time PCR (qRT-PCR) analysis was conducted with 1 ng of cDNA in a 20 μL reaction volume using iTaq™ SYBR^®^ Green Supermix and ROX dye (Bio-Rad, Hercules, CA, USA). The gene-specific primers are listed in Supplementary Table S1. The qRT-PCR cycles consisted of 95 °C for 10 min followed by 40 cycles of 95 °C for 20 s, 58 °C for 20 s, and 72 °C for 25 s. Fluorescence was recorded after the last step of every cycle. Three replicates were performed per sample. Amplification, data processing, and detection were performed using the CFX96 Real-Time PCR Detection System (Bio-Rad). Detected quantification cycle (cq) values were examined using the 2^−ΔCT^ method to reveal changes in gene expression.

### 2.8. Statistical Analysis

For statistical analysis, Student’s *t*-test was performed and *p* < 0.05 was considered significant. Data are expressed as means ± standard deviation (SD) of three biological replicates.

### 2.9. Data Depositon

Raw data files were submitted to the sequence reads archive (SRA), NCBI database (SRR15858696, SRR15858697, SRR15858698, SRR15858699, SRR15858700, SRR15858701).

## 3. Results

### 3.1. Promotion of Growth and Secondary Metabolites in GI Knockdown Chinese Cabbage

**Figure 1:**
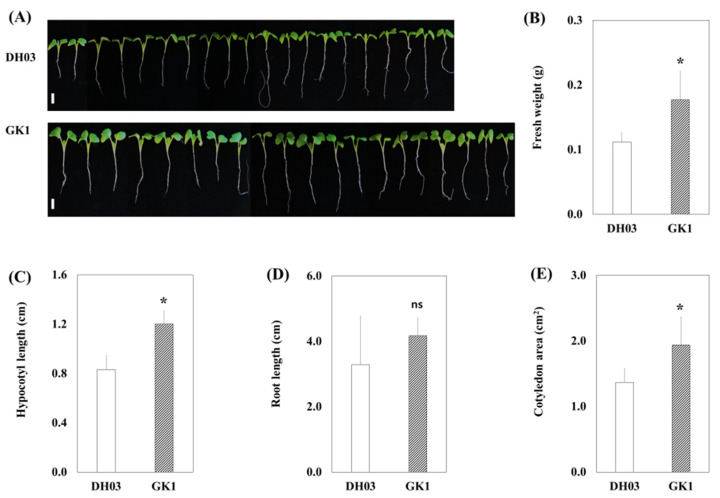
Phenotypes of *B. rapa* sprouts of DH03 and GK1 grown under 16/8 h light/dark conditions. Twenty representative images of 8-day-old Chinese cabbage sprouts are shown. Bar = 1 cm. (**A**), Fresh weight (**B**), hypocotyl length (**C**), root length (**D**), and cotyledon area, and (**E**) of 8-day-old Chinese cabbage sprouts. Each value is the mean of three biological replicates, and error bars indicate the standard deviation (SD). * *p* < 0.05 and ns = not significant compared to the negative control sprouts (DH03).

A GI knockdown mutant (hereafter referred to as GK1) that showed strong tolerance to salt stress and high suppression of GI mRNA and protein levels was obtained [18]. We observed that 8-day sprouts of GK1 transgenic plants were significantly larger than the wild-type, DH03 (Figure 1A). The fresh weight of GK1 seedlings was 0.2 g/20 plants, twofold higher than the 0.1 g/20 plants of DH03 seedlings (Figure 1B). Additionally, the hypocotyl length, root length, and cotyledon area of GK1 seedlings were 1.2, 4, and 1.9 cm^2^, respectively, which were 1.3–1.5-fold those of DH03 seedlings (0.8, 3, and 1.4 cm^2^) (Figure 1C–E).

### 3.2. Primary and Secondary Metabolite Contents

**Figure 2:**
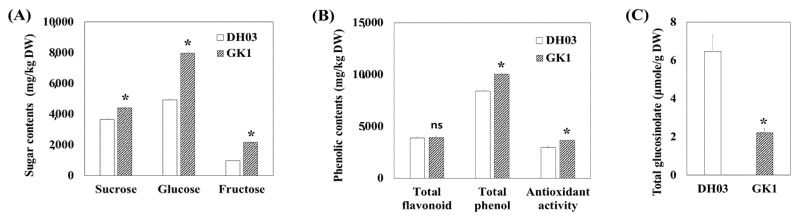
Primary and secondary metabolite contents in DH03 and GK1 Chinese cabbage sprouts. Primary metabolites (**A**): sucrose, glucose, and fructose, secondary metabolites (**B**): total phenols, total flavonoids, and GSLs (**C**) in DH03 and GK1 Chinese cabbage sprouts. Each value is the mean of three biological replicates, and error bars indicate the standard deviation (SD). * *p* < 0.05 and ns = not significant compared to the negative control sprouts (DH03).

The sucrose, glucose, and fructose levels of GK1 transgenic plants were higher than DH03 (Figure 2A). In particular, glucose and fructose levels were substantially increased in GK1 seedlings, from 4933 to 7963 mg/kgDW and 989 to 2177 mg/kgDW, respectively, compared to the wild-type. Additionally, secondary metabolite contents were changed in GK. The total phenol content and antioxidant activity were 1.2-fold higher in GK1 than DH03, but the total flavonoid contents were similar (Figure 2B). GSL is a major secondary metabolite in *Brassica* crops. The expression of GSL biosynthetic genes showed a daily rhythm and typically peaked 4 h after sunrise [36]. Therefore, we measured the GSL contents in wild type and GK1 Chinese cabbage. The total GSL level decreased by two-third (2.22 μmole/gDW) in the GK1 transgenic line compared to 6.46 μmole/gDW in the wild type, DH03 (Figure 2C).

### 3.3. Transcriptomic Analysis

**Figure 3:**
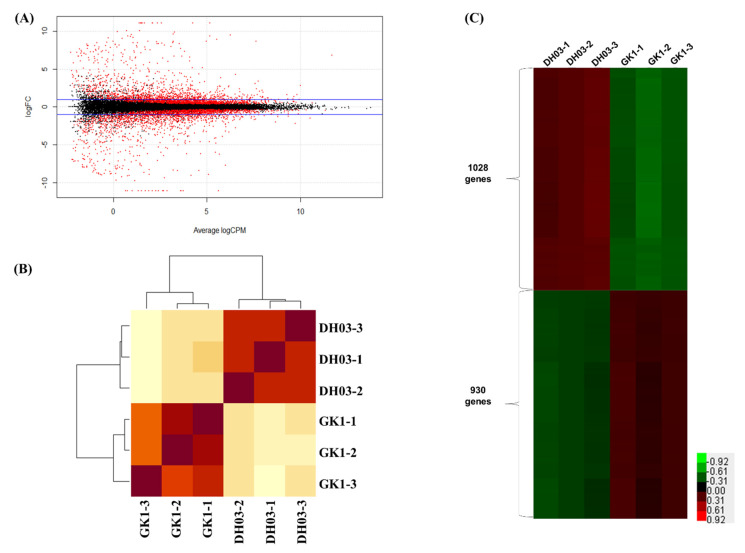
Statistical analysis of differentially expressed genes across samples. (**A**) MA plots showing pairwise comparisons of transcript levels between DH03 and GK1. *Y*-axis: log2 fold change (logFC) between two samples; *X*-axis: log2 average count normalized to the size factor. Red dots: transcripts with an absolute log_2_ fold change ≥ 1; black dots: transcripts with an absolute log_2_ fold change < 1. (**B**) Clustered heatmap showing the Pearson correlation matrix for pairwise sample comparisons. The color key was adjusted based on the log2-centered values to show differences. Dendrograms show distances between repeat samples. (**C**) Number of significantly expressed genes (FDR *p* < 0.05) between DH03 and GK1. A total of 1028 downregulated and 930 upregulated genes in GK1 compared to DH03 are represented.

**Figure 4:**
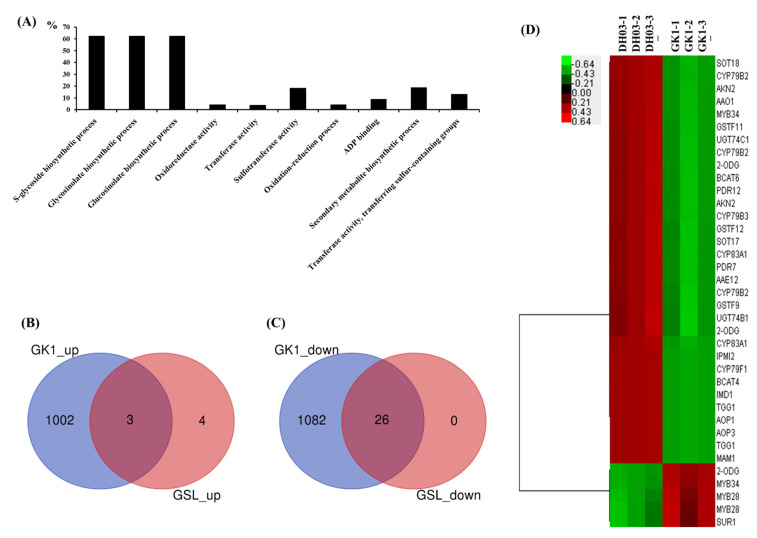
Expression of GSL pathway-related genes. GO term enrichment analysis indicates that GSL-related categories were the most significantly downregulated DEGs in GK1 (red bars). ShinyGO (v. 0.61) was employed for GO analysis (http://bioinformatics.sdstate.edu/go/, accessed on 20 August 2021). The percentage (%) on the *y*-axis is calculated as follows: % = (number of genes in DEG list ÷ total corresponding pathway genes) × 100 (**A**). Venn diagram showing GSL pathway genes among 930 DEGs upregulated ≥ 2-fold (**B**) and 1028 DEGs downregulated ≥ 2-fold in GK1 (**C**). Heatmap of GSL pathway genes among DEGs between DH03 and GK1 (**D**).

We compared the genome-wide transcriptome of GK1 and the wild-type, DH03. We isolated total RNA from the aerial parts of wild-type and GK1 plants at 4 h after sunset, and synthesized cDNA libraries for RNAseq, generating 235,146,532 raw paired reads. After filtering out low-quality and unpaired reads, 220,681,405 high-quality paired reads were used for mapping of the *B. rapa* transcriptome. They showed a mapping rate of >92% on *B. rapa* reference transcript sequences (Table 1). Correlation heatmap analyses showed distinct clustering of DH03 and GK1 samples (Figure 3A), indicating that RNA-seq libraries were well prepared and sequenced. We isolated differentially expressed genes (DEGs) between DH03 and GK1 samples based on pairwise sample comparisons (Figure 3B). In total, 5232 genes were selected as “high-confidence DEGs” (*p* < 0.05) (15.8% of the 33,133 annotated genes) in pairwise sample comparisons. Among them, 1958 DEGs (5.9%) were isolated as significant DEGs (>2-fold change) between DH03 and GK1, including 1028 downregulated and 930 upregulated genes in GK1 compared to DH03 (Figure 3C). To investigate the biological functions of the DEGs, a GO analysis was performed of high-confidence (up- or down-regulated) DEGs (>2-fold change and *p* < 0.05) between GK1 and DH03. Unexpectedly, no obvious categories were identified in the 930 upregulated DEGs in GK1 using ShinyGO v. 0.66 (http://bioinformatics.sdstate.edu/go/, accessed on 20 August 2021). However, GO term analysis using 1028 downregulated DEGs in GK1 yielded the following top 10 functional categories: “S-glycoside biosynthetic process”, “Glycosinolate biosynthetic process”, “Glucosinolate biosynthetic process”, “Oxidoreductase activity”, “Transferase activity”, “Sulfotransferase activity”, “Oxidation-reduction process”, “ADP binding”, “Secondary metabolite biosynthetic process”, and “Transferase activity, transferring sulfur-containing groups” (Figure 4A). Interestingly, glucosinolate-related categories were abundant in the list of downregulated genes in GK1. This suggests that a clock component, BrGI, affects GSL metabolism in Chinese cabbage.

### 3.4. Expression of Genes Related to GSL

**Figure 5:**
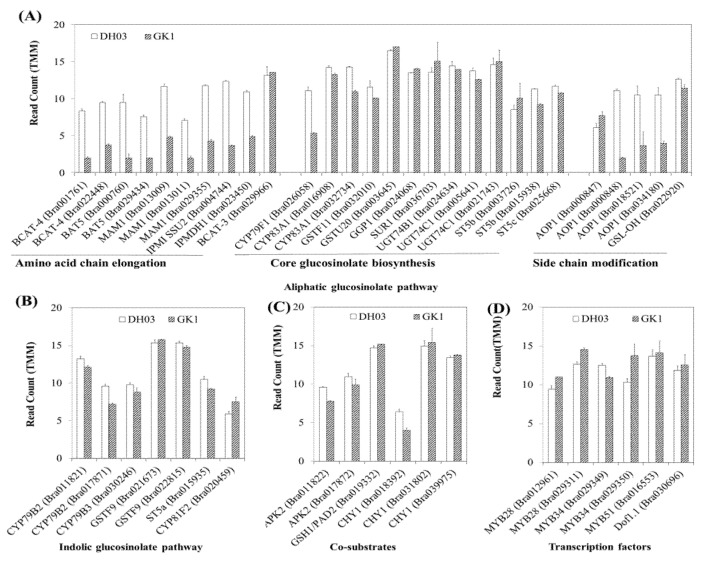
Expression of candidate genes related to GSL pathways between DH03 and GK1. Orthologs of Arabidopsis genes in the BRAD genome database (http://brassicadb.cn/, accessed on 20 August 2021) were screened in *B. rapa* and significantly expressed genes (FDR *p* < 0.05) were identified (Supplementary Table S2). Candidate genes were classified according to the step of the GSL biosynthesis pathway. Aliphatic GSL pathway (**A**), indolic GSL pathway (**B**), co-substrates (**C**), and transcription factors (**D**).

Because the GO analysis of downregulated genes in GK1 suggested an abundance of glucosinolate-related categories, we searched and extracted 138 GSL pathways from the BRAD genome database (http://brassicadb.cn/, accessed on 20 August 2021). Among the GSL pathway genes, high-confidence DEGs (FDR > 0.05) were isolated by Venn analysis (Figure 4B,C, and Supplementary Table S3). Thirty-five GSL pathway genes were in the significantly changed DEGs dataset (>2-fold change). The majority of the GSL pathway genes (30 genes) were downregulated in GK1 (Figure 4C,D), although five were upregulated in GK1 compared to DH03 (Figure 4B,D). A group of MYB transcription factors (TFs) play a role in the regulation of GSL metabolism [40,46,47,48,49]. One *MYB34* (*Bra029349*) was downregulated (Figure 4D) and four MYB TFs (*Bra029350*, *Bra029311*, and *Bra012961*) were upregulated in GK1. GSL compounds are mostly synthesized by two major biosynthetic pathways (aliphatic and indole) in Brassicaceae family plants. The aliphatic GSL pathway has three biosynthetic stages (amino acid side elongation, core structure formation, and secondary modification), whereas the indole GSL pathway has two (core structure formation and secondary modification). To evaluate the transcriptional pattern of GSL pathway genes, we compared the RNA-seq read counts of individual GSL pathway genes between DH03 and GK1 (Figure 5A–D). In the aliphatic GSL pathway, many genes related to amino acid chain elongation and side chain modification were significantly decreased in GK1, suggesting that BrGI promotes amino acid side elongation and secondary modification (Figure 5A). However, most genes involved in core structure formation, which overlapped with the indole GSL biosynthetic pathway, did not differ significantly between DH03 and GK1 (Figure 5A,B). Additionally, the expression levels of co-substrate and TF-related genes in the GSL pathway were similar between the two genotypes (Figure 5C,D). Therefore, BrGI facilitates biosynthesis of aliphatic GSLs by activating genes involved in amino acid side elongation and secondary modification, but not core structure formation. The transcript levels of individual indole GSL pathway genes did not differ between the two genotypes (Figure 7B).

**Figure 6:**
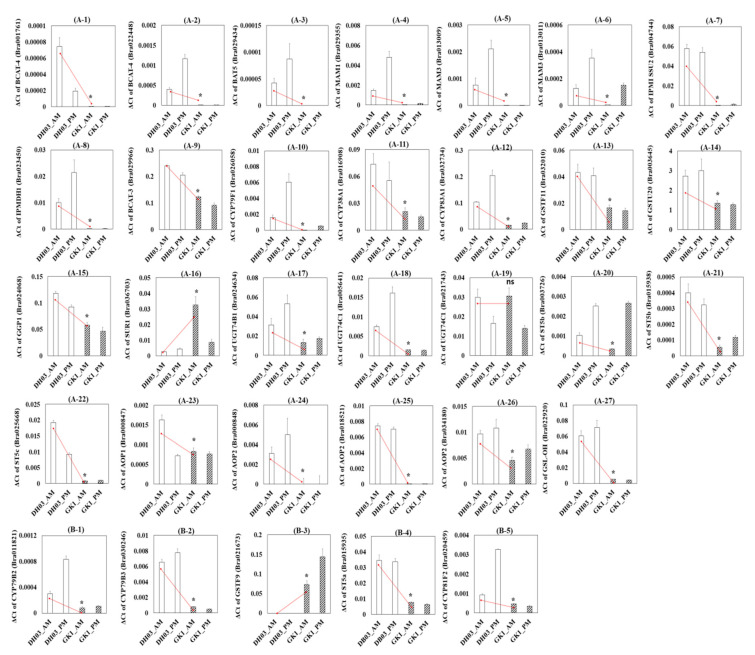
RT-qPCR validation of RNA-seq data. For RT-qPCR of the diurnal expression of GSL synthesis-related genes in DH03 and GK1 Chinese cabbage sprouts, samples were collected at 10 a.m. and 5 p.m. in liquid nitrogen and stored at −80 °C prior to lyophilization. The DEGs detected by RT-qPCR showed the same trend as those identified by RNA-seq (Figure 5). Genes in the aliphatic GSL pathway (**A-1**–**A-27**) and indolic GSL pathway (**B-1**–**B-5**). Each value is the mean of three biological replicates, and error bars indicate the standard deviations (SDs). * *p* < 0.05 compared to the negative control (DH03). CYP79B2c (Bra017871), GSTF9 (Bra022815), and BAT5 (Bra000760) were not detected.

To confirm the RNA-seq data (Figure 4D and Figure 5), we performed a qRT-PCR analysis of the two genotypes. Transcript levels of individual aliphatic and indole GSL pathway genes were measured in the morning (4 h after sunrise) and evening (4 h before sunset) in DH03 and GK1. Similar to the RNA-seq read counts, a majority of aliphatic GSL pathway genes related to amino acid side elongation and secondary modification were significantly reduced in GK1, in both the morning and evening (Figure 6). For example, 25 of 27 (93%) aliphatic GSL pathway genes showed reduced transcript levels in GK1 compared to DH03. We also measured the transcript levels of the seven differentially regulated indole GSL pathway genes Four indole GSL genes (*CYP79B2*, *CYP79B3*, *ST5a*, and *CYP81F2*) were downregulated in GK1, whereas one indole GSL gene (*GSTF9*) was upregulated in GK1 at both time points (Figure 6). Expression of CYP79B2c (Bra017871) and GSTF9 (Bra022815) was not detected. Collectively, these results confirmed that normalized RNA-seq count data accurately reflect the endogenous transcript levels of GSL pathway genes between DH03 and GK1. Reduced GI expression suppressed the expression of GSL pathway genes.

### 3.5. GSL Compounds in GK1

**Figure 7:**
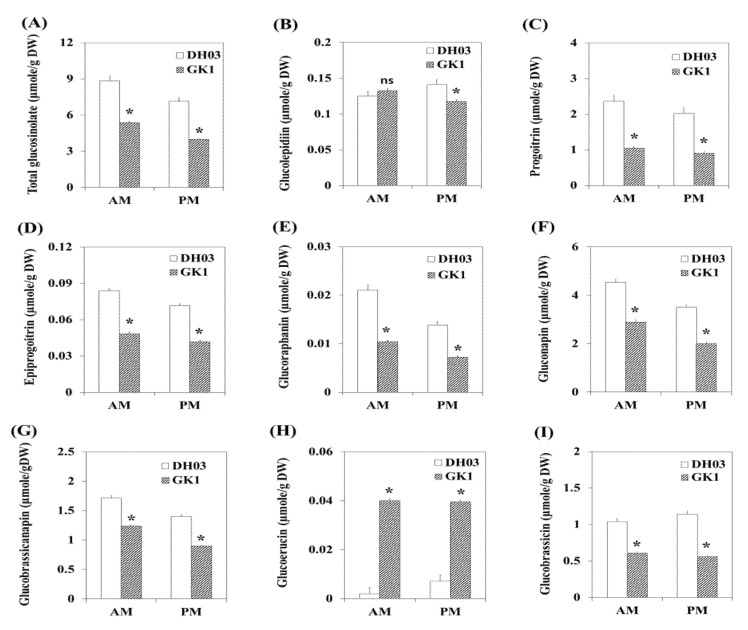
Diurnal accumulation of GSL in DH03 and GK1 grown under 16/8 h light/dark conditions. Samples were collected at 10 a.m. and 5 p.m. in liquid nitrogen and stored at −80 °C prior to lyophilization. Total GSLs (**A**), glucolepidiin (**B**), progoitrin (**C**), epiprogoitrin (**D**), glucoraphanin (**E**), gluconapin (**F**), glucobrassicanapin (**G**), glucoerucin (**H**), and glucobrassicin (**I**). Each value is the mean of three biological replicates, and error bars indicate the standard deviations (SDs). * *p* < 0.05 compared to the negative control (DH03). ns = not significant compared to the negative control sprouts (DH03).

Because many GSL pathway genes were downregulated in GK1, we measured the amounts of endogenous GSL compounds in the morning (4 h after sunrise) and evening (4 h before sunset). Seven aliphatic GSLs (glucolepidiin, progoitrin, epiprogoitrin, glucoraphanin, gluconapin, glucobrassicanapin, and glucoerucin) and one indole GSL compound (glucobrassicin) were detected (Figure 7). All GSL compounds except glucolepidiin and glucoerucin were significantly reduced in GK1 compared to DH03 (Figure 7B–I). Therefore, BrGI is required for GSL biosynthesis in Chinese cabbage. This is in the line with the RNA-seq and RT-qPCR results. The *BrGI* circadian clock gene is involved in the regulation of GSL metabolism in Chinese cabbage and promotes the biosynthesis of GSL compounds. Modulation of BrGI might be a novel strategy to control the endogenous levels of GSLs in Chinese cabbage plants.

## 4. Discussion

### 4.1. Reduced Expression of GI Alters the Main Traits of Crop Plants

The GI clock gene plays various roles in plant biological processes [23,24]. As a clock regulator, GI interacts with several clock genes; its expression peaks at specific times of day depending on the plant species and light period [18,23], and is involved in stabilizing the ZTL blue-light photoreceptor [50,51]. GI functions downstream of PhyA-B and shows changing cotyledon shapes or hypocotyl phenotypes under different light qualities [52,53]. Furthermore, GI affects hypocotyl growth by interacting with SPINDLY (SPY), which is related to gibberellin signaling and the auxin-responsive protein, SMALL AUXIN UPREGULATED RNA 22 (SAUR22) [54,55]. Transgenic poplar plants with down-regulated expression of PagGI genes exhibited increased biomass and auxin-overproduction morphological phenotypes, characterized by increased transcript levels of early auxin-response genes [29]. Under long-day (16 h day/8 h night) conditions, decreased COP1 induces auxin synthesis by activating PIF4, leading to root and shoot elongation [56]. The phenotypes of transgenic poplar plants might be altered by light modulation of auxin signaling systems, including IAA biosynthesis [57]. In this study, 7-day-old seedlings of *GI*-knockdown Chinese cabbage showed increased biomass. The fresh weight, hypocotyl length, root length, and cotyledon area increased 2–1.3-fold compared to wild-type plants (Figure 1A–E). The light-signaling gene, COP1, was downregulated and the auxin-signaling gene, IAA19 (Bra001598) [56,58], was upregulated in GK1 (Supplementary Figure S1B,D,F). Moreover, GK1 plants were larger and heavier after heading than DH03 plants.

Studies of Arabidopsis *GI*-mutants and RNA interference of one *GI* paralogue, PhGI1 in Petunia × hybrida, suggest a function for *GI* in chloroplast biogenesis and chlorophyll accumulation [59,60]. Additionally, *GI* is required for the response of the circadian clock to sucrose [61]. The starch content was increased by *GI*-null alleles in Arabidopsis [62], and field-grown rice plants carrying the *OsGI* null mutation showed significantly increased leaf sucrose and starch contents at most time points [63]. The sucrose, glucose, and fructose contents increased in GK1 plants. The diurnal cycles were conserved in DH03 and GK1. However, the levels of these primary metabolites in GK1 plants were increased 4 h after sunrise (Supplementary Figure S2). Wild-type plants under an abnormal photoperiod, and clock-mutant plants under a normal photoperiod, underwent reduced growth due to excess starch degradation, suggesting that sugar synthesized during the day was consumed at night to maintain and improve plant growth [20]. In GK1, the diurnal rhythm was conserved but several clock orthologous genes, CCA1s, LHYs, PRR1s and PRR5s (including *GI*) showed unchanged expression levels compared to the wild-type (Supplementary Figure S1A,C,E). Although the relationship between *GI* and carbohydrate metabolism is unclear, changing transcript levels might induce starch degradation at night and high levels of monosaccharides in the morning, thereby improving seedling growth (Figure 1A–E and Figure 2A, and Supplementary Figure S2). Although the relationship between downregulation of *GI* and increased biomass and primary metabolite levels is unclear, the results suggested that *GI* could be used to improve crop productivity without changing their functional and nutritional contents.

### 4.2. Reduced Expression of GI Could Affect the GSL Pathway

GSL accumulation could re-entrain the plant’s endogenous clock, and GSL genotypes altered the periodicity of the clock output in a manner unrelated to the photochemical state of photosystem II [17]. GSLs are sulfur-containing secondary metabolites, and sulphate assimilation is controlled by the diurnal rhythm and light. Therefore, GSL levels during the day were higher than at night and GSL synthesis genes were controlled at least in part by the LONG HYPOCOTYL5 (HY5) transcription regulator [33]. In *B. rapa*, the expression of most GSL genes increased to a peak in the first 4 h after sunrise (ZT4), inducing BCAT4 and MAM1 (early side chain elongation) and AOP2 (secondary modification of side chains) [36]. Myeloblastosis (MYB) proteins are a large family of TFs in plants that play major regulatory roles in many biological processes [46,47]. Diverse environmental stimuli, including wounding, pathogens, insect herbivores, light, and nutrition, regulate GSL metabolism through MYB TFs [49,64]. Fourteen functional R2R3-MYB genes are involved in GSL biosynthesis in *B. rapa* [40]. Although the GSL level decreased in GI knockdown plants, the expression levels of TFs, including the MYB family, were unchanged (Figure 5D and Figure 6). BCAT4 (Bra001761 and Bra022448), MAM1 (Bra013009, Bra013011, and Bra029355) (early steps of side chain elongation) and AOPs (Bra000848, Bra018521, and Bra034180) (side chain modification) were downregulated in GK1 but genes related to core GSL biosynthesis involved in both aliphatic and indole GSLs were expressed similarly in DH03 and GK1. Furthermore, in indolic GSL biosynthesis, CYP79B2 (Bra011821 and Bra017871) and CYP79B3 (Bra030246) (Arabidopsis cytochrome P450 enzymes that convert tryptophan into indole-3-acetaldoxime (IAOx), the common precursor of auxin, camalexin, and indolic GSLs) [65,66] were not significantly downregulated in GK1. These patterns might lead to higher expression of IAA19 (Bra001598) in GK1 seedlings (Supplementary Figure S1B,D,F). The regulation of indolic GSLs by glucose signaling through MYB34, MYB51, and MYB122 is distinct from that of aliphatic GSLs [64]. Although the sugar contents (including glucose) were higher in GK1 than DH03, both indolic and aliphatic GSLs compound levels in GK1 decreased compared to those of DH03 (Figure 2A,C). These results provide new insight into the molecular mechanisms involving *GI* and clock genes that underlie GSL biosynthesis. However, more studies are needed to better understand the interactions between clock regulators and GSL.

### 4.3. Clock Genes Could Be Modified to Improve Crop Productivity and Quality

GSLs and their breakdown products not only contribute to the distinctive flavor and aroma of cruciferous vegetables, but are also involved in plant defense mechanisms, auxin homeostasis and cancer prevention in humans [59]. Research is increasingly focusing on the regulatory mechanisms of GSL biosynthesis, and on the feasibility of customizing GSL profiles by molecular breeding [67,68,69]. Coordination of plant circadian rhythms with the external environment provides growth and reproductive advantages to plants, as well as enhanced resistance to insects and pathogens. We evaluated whether clock components regulate GSLs. Although further studies are needed on the relationship between *GI* and GSLs biosynthesis, changing the expression of *GI* could alter the total GSL content and composition. Some aliphatic GSLs, like progoitrin, are toxic to animals and their bitter taste reduces the palatability of kimchi. In this study, the total GSL levels and most components (including progoitrin) significantly decreased (Figure 7B–I). Therefore, circadian clock genes could be used to improve the quality of crop plants.

## Figures and Tables

**Table 1:** Statistical analysis of *Brassica rapa* RNA sequencing (RNA-seq) reads. R1–3: three replications.

Sample Name	Read-Pairs	Both Surviving	Overall Alignment Rate	Concordant Zero	Concordant Pair Alignment	Multiple Alignment
DH03_R1	39,266,003	36,514,950 (92.99%)	91.71%	12.79%	84.82%	2.39%
DH03_R2	41,520,758	38,946,152 (93.80%)	91.87%	12.46%	85.04%	2.49%
DH03_R3	43,990,750	41,288,526 (93.86%)	91.92%	12.54%	84.99%	2.47%
GK1_R1	39,987,718	37,533,690 (93.86%)	91.95%	12.70%	84.69%	2.61%
GK1_R2	31,435,216	29,695,080 (94.46%)	92.16%	12.32%	85.34%	2.34%
GK1_R3	38,946,087	36,703,007 (94.24%)	92.03%	12.55%	84.90%	2.55%

## Supplementary Materials

The following are available online at https://www.mdpi.com/article/10.3390/genes12111664/s1, Figure S1: The expression of clock genes ((A), (C), and (E)) and light signal genes ((B), (D), and (F)), Figure S2: Diurnal accumulation of primary ((A), (B), and (C)) and secondary metabolite ((D), (E), and (F)) contents and in DH03 and GK1 Chinese cabbage sprouts grown in 16-/8-h light/dark condition; Table S1: Primer lists used for qRT- PCR analysis, Table S2: The GSL-related genes showing differential expression in GK1 line compared to DH03 wild type, Table S3: The circadian clock genes showing differential expression in GK1 line compared to DH03 wild type, Table S4: The light regulation related genes showing differential expression in GK1 line compared to DH03 wild type, Table S5: GO analysis of 2 fold down regulated transcriptome in GK1.
