# Supplementary material for: The BrGI Circadian Clock Gene Is Involved in the Regulation of Glucosinolates in Chinese Cabbage
Source: Genes (Basel). 2021 Oct 22;12(11):1664. doi: 10.3390/genes12111664 (PMC8621042; doi:10.3390/genes12111664)
Supplement: Supplementary file 1 [file genes-12-01664-s001.zip › Supplementary Figure S1-2.pptx]

## Slide 1
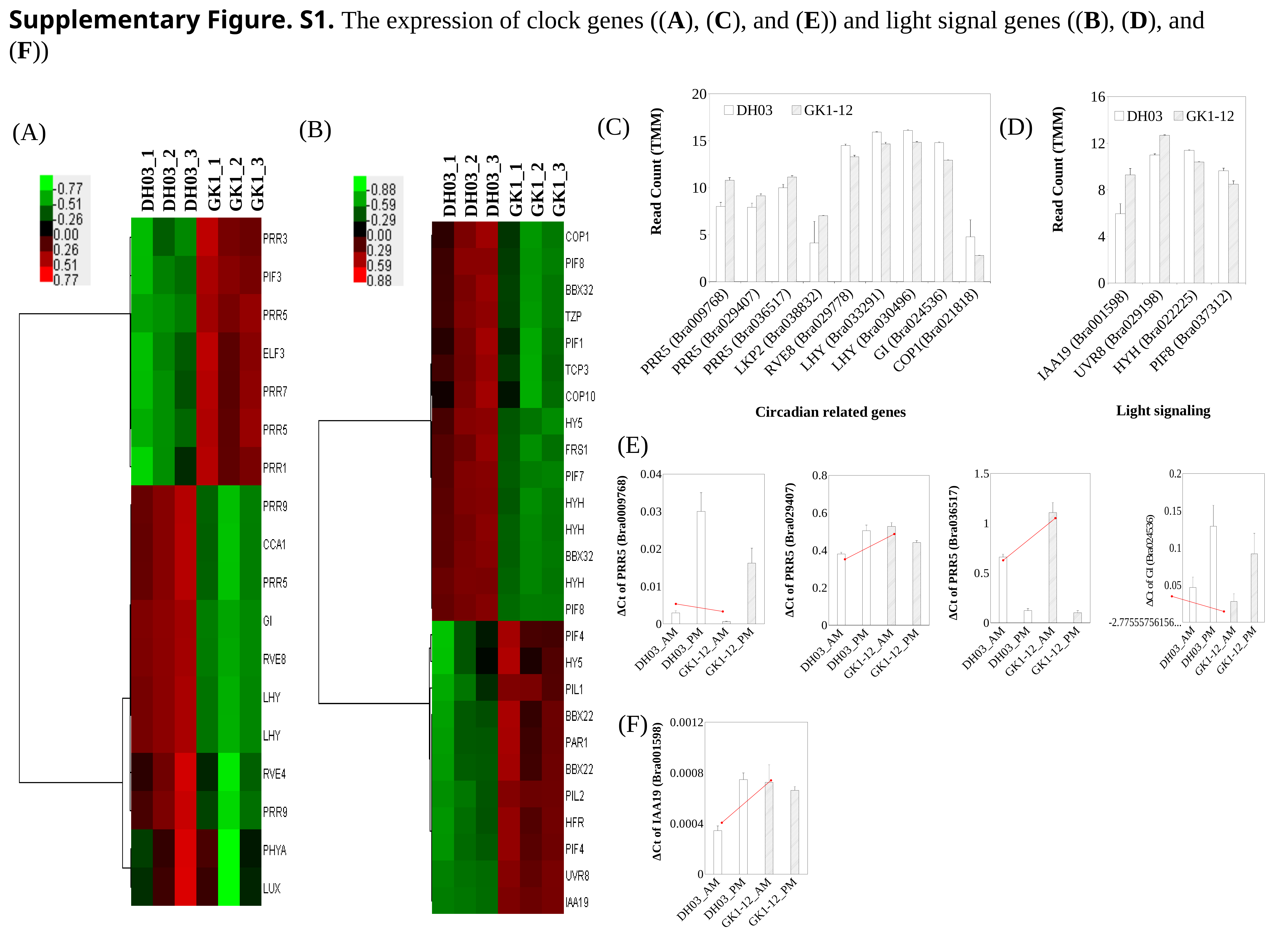

Supplementary Figure. S1. The expression of clock genes ((A), (C), and (E)) and light signal genes ((B), (D), and (F))
### Chart
| Category | DH03 | GK1-12 |
|---|---|---|
| PRR5 (Bra009768) | 8.021477573577023 | 10.801017841072769 |
| PRR5 (Bra029407) | 7.932599265913059 | 9.149935849537185 |
| PRR5 (Bra036517) | 9.994703985867883 | 11.147513063060666 |
| LKP2 (Bra038832) | 4.124179164609404 | 7.022819297294693 |
| RVE8 (Bra029778) | 14.505032191983402 | 13.295334629618033 |
| LHY (Bra033291) | 15.924548933808866 | 14.678429827541466 |
| LHY (Bra030496) | 16.0926443493227 | 14.8399025726956 |
| GI (Bra024536) | 14.7943599902286 | 12.930173250652198 |
| COP1(Bra021818) | 4.772418448075396 | 2.80240314452169 |
### Chart
| Category | DH03 | GK1-12 |
|---|---|---|
| IAA19 (Bra001598) | 5.943255833973396 | 9.28739397611784 |
| UVR8 (Bra029198) | 10.994003844929201 | 12.677150762896298 |
| HYH (Bra022225) | 11.399189062960966 | 10.392939875786134 |
| PIF8 (Bra037312) | 9.647137814407847 | 8.491994026440361 |(D)
(C)
(B)
(A)
DH03_1
DH03_2
DH03_3
GK1_1
GK1_2
GK1_3
DH03_1
DH03_2
DH03_3
GK1_1
GK1_2
GK1_3
(E)
### Chart
| Category | |
|---|---|
| DH03_AM | 0.6597539553864449 |
| DH03_PM | 0.1235642525441118 |
| GK1-12_AM | 1.107008781595307 |
| GK1-12_PM | 0.10176640845071201 |
### Chart
| Category | |
|---|---|
| DH03_AM | 0.04676401713528785 |
| DH03_PM | 0.1290638503488882 |
| GK1-12_AM | 0.027786885270074835 |
| GK1-12_PM | 0.09183005957371815 |
### Chart
| Category | |
|---|---|
| DH03_AM | 0.0028927686568097025 |
| DH03_PM | 0.030046345390082885 |
| GK1-12_AM | 0.0006209077960868171 |
| GK1-12_PM | 0.016213432174371035 |
### Chart
| Category | |
|---|---|
| DH03_AM | 0.37980566605890026 |
| DH03_PM | 0.5046424006059378 |
| GK1-12_AM | 0.527289314758007 |
| GK1-12_PM | 0.4413514981453286 |
### Chart
| Category | |
|---|---|
| DH03_AM | 0.0003434661520792235 |
| DH03_PM | 0.0007465278734772128 |
| GK1-12_AM | 0.0007247404605911087 |
| GK1-12_PM | 0.0006613233594495869 |(F)

## Slide 2
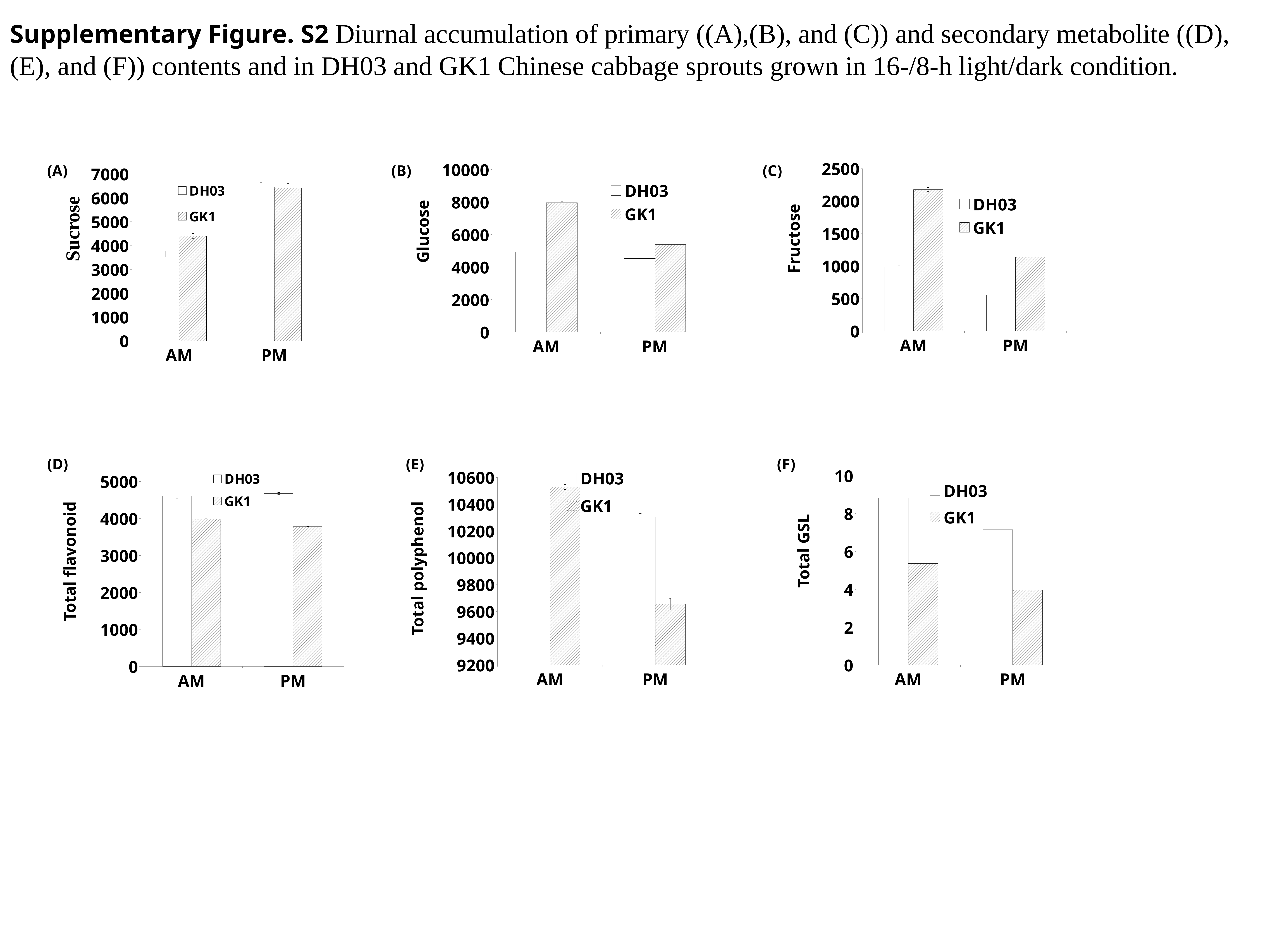

Supplementary Figure. S2 Diurnal accumulation of primary ((A),(B), and (C)) and secondary metabolite ((D),(E), and (F)) contents and in DH03 and GK1 Chinese cabbage sprouts grown in 16-/8-h light/dark condition.
(A)
### Chart
| Category | DH03 | GK1 |
|---|---|---|
| AM | 989.1487944993531 | 2177.4817163616535 |
| PM | 554.8939556515093 | 1140.2612017222398 |(B)
(C)
### Chart
| Category | DH03 | GK1 |
|---|---|---|
| AM | 4933.407669194924 | 7963.841696604752 |
| PM | 4535.522388324213 | 5390.569123896715 |
### Chart
| Category | DH03 | GK1 |
|---|---|---|
| AM | 3654.838936811851 | 4401.994949282886 |
| PM | 6448.730821036213 | 6402.129160848269 |Sucrose
Glucose
Fructose
(D)
(E)
(F)
### Chart
| Category | DH03 | GK1 |
|---|---|---|
| AM | 10252.08333333333 | 10528.124999999998 |
| PM | 10306.249999999998 | 9653.125 |
### Chart
| Category | DH03 | GK1 |
|---|---|---|
| AM | 4609.5288095550595 | 3980.9055906897324 |
| PM | 4675.451695440693 | 3783.6607486780827 |
### Chart
| Category | DH03 | GK1 |
|---|---|---|
| AM | 8.841794994811716 | 5.366847842122898 |
| PM | 7.151322156895624 | 3.9709895827765647 |Total GSL
Total flavonoid
Total polyphenol
