# Supplementary material for: The BrGI Circadian Clock Gene Is Involved in the Regulation of Glucosinolates in Chinese Cabbage
Source: Genes (Basel). 2021 Oct 22;12(11):1664. doi: 10.3390/genes12111664 (PMC8621042; doi:10.3390/genes12111664)
Supplement: Supplementary file 1 [file genes-12-01664-s001.zip › supplementary Table S1~S4.pdf]

Supplementary Table S1. Primer lists used for qRT- PCR analysis

Brassica ID	Arabidopsis ID	Gene name	Primer sequence (5'→3')
Bra001761	At3g19710	BCAT-4	Forward: AAGTATTGTTCCTTACGCTG<br>Reverse: AAGACGAAGGGCGTTTTGG
Bra022448			Forward: CCGGGGAGAGGAACATTGTA<br>Reverse: CACTTGGAAAAGCACGACGA
Bra000760	At4g12030	BAT5	Forward: CGCAACAGCTTCTCACCTT<br>Reverse: TTATAGATCTCATTTGTGTC
Bra029434			Forward: ACTTTTTAACTGGTTTGCTC<br>Reverse: CGTCTGCCAAAAAATAAGAAT
Bra013009	At5g23010	MAM1	Forward: TCATACGTGATGGATGGTGT<br>Reverse: TCTTCTGGAGATAAGATCTC
Bra013011			Forward: AGCAGCAACATGTGCGTGTA<br>Reverse: CGCAGGGAAACCAACTTCCA
Bra029355			Forward: CGAACAAGCTCCCAGACAAG<br>Reverse: GGCGATGGTTTGGATGGTTT
Bra004744	At2g43100	IPMI SSU2	Forward: TCCCATGGATCTGGCTCTAC<br>Reverse: GTCAGCTGGGATGATCTGGT
Bra023450	At5g14200	IPMDH1	Forward: GGGAATCAAGACCAACGAAA<br>Reverse: CATCCAACACATTGGCTTTG
Bra029966	At3g49680	BCAT-3	Forward: TGAGATGTGGTGCTGAGAGG<br>Reverse: GCAGGAGCAAGACCAAGAAC
Bra026058	At1g16410	CYP79F1	Forward: TCGTCAAGATACCACCCTCG<br>Reverse: ACGACACGAAACGCAATTCA
Bra016908	At4g13770	CYP83A1	Forward: CAGAACCTTAACCCACAACGGT<br>Reverse: TCCGAAAAGTTGACGTCTT
Bra032734			Forward: TCGCGAACCTTCTCCTCAA<br>Reverse: TTACTTGCTCACTTTCTCGG
Bra032010	At3g03190	GSTF11	Forward: GAAAAGGGCATCGAGTTTGA

			Reverse: CCTCGATATGGCTCGTGATT
Bra003645	At1g78370	GSTU20	Forward: AGTCGCGCTGAGAGAGAAAG<br>Reverse: ACCAAGCCTCGTCCACATAC
Bra024068	At4g30530	GGP1	Forward: AGTTCCCCGACGAGAAAGAT<br>Reverse: TCTGGTGACCAAAGCAGATG
Bra036703	At2g20610	SUR1	Forward: GTCTCGAGGTTCGCAAGTTC<br>Reverse: GTCTCTGCGACCTTTTGGAG
Bra024634	At1g24100	UGT74B1	Forward: CGAATCCTTCAAGCTCCACG<br>Reverse: GAAGGACAGAGCAAACGGTG
Bra005641<br>Bra021743	At2g31790	UGT74C1	Forward: AGGAGATTACAAGATGCGTT<br>Reverse: ATCATAGCAACAAATTCATC<br>Forward: ATGAGTGAAGCAAGCGGTCA<br>Reverse: GACAGGAAACCGTCGTAGAT
Bra003726<br>Bra015938	At1g74090	ST5b	Forward: GATTGGCTGAGTTCATGGGT<br>Reverse: GTGTCGTTTGTATAAGGAGA<br>Forward: TTACCAAACACTAATCTCCA<br>Reverse: TACAGACGAAGATGTCGTCG
Bra025668	At1g18590	ST5c	Forward: GAAGGCAACACGCTCTTCTC<br>Reverse: TCCTTGCTGTGACCTCTCCT
Bra000847	At4g03070	AOP1	Forward: TTCATCGTCATGGTTGGAGA<br>Reverse: CACGTGGATGTTCTTCATCG
Bra000848<br>Bra018521<br>Bra034180	At4g03070	AOP2	Forward: AACCTTGTCATTGTCACCAGT<br>Reverse: CAAGAGTACCAGCGAAAGGAT<br>Forward: ACAAGAGTATCAGCGAAACAATCC<br>Reverse: ACTAGTATCAGCAACAACATTGGC<br>Forward: AAGAGTATGAGCGAAACGATCCAG<br>Reverse: CAGCATCAGCGTTAGCAGTTG
Bra022920	At2g25450	GSL-OH	Forward: ATTGCGCAAAGGGTTTATTG<br>Reverse: TAAGAGCACCCGGAGTAGGA
Bra011821<br>Bra017871	At4g39950	CYP79B2	Forward: TTCAAGAAAATGAGGAAAGT<br>Reverse: AACGTTCTTGTTCCGAACAT<br>Forward: ATGAACACTCTTACCTCAAACT

			Reverse: TTAGGATTAGTGATCATTTTC
Bra030246	At2g22330	CYP79B3	Forward: ACCGCTGATGAAATCAAACC<br>Reverse: TTCTTTGCCGACGACTCTTT
Bra021673	At2g30860	GSTF9	Forward: TGTCTACGAGGCACATCTCG<br>Reverse: AAGCAGGACGGCTGCTAATA
Bra015935	At1g74100	ST5a	Forward: GATCCTTTGCCCTTTGTGAA<br>Reverse: CGGTCCTCCCTCTCCTTATC
Bra020459	At5g57220	CYP81F2	Forward: AATAGTGGTGCAAGCCATCC<br>Reverse: GCATTGTGTTGCTCTCTCCA
Bra009768	At5g24470	PRR5	Forward: CCCGAAGATATCTCTCAGGG<br>Reverse: TATCCGGAGATCGAAGGAAG
Bra029407			Forward: TCCGAAAATCTCGCTAAGAG<br>Reverse: GAGCGTATCCAGAGATTGAAGG
Bra036517			Forward: CCTAAAATCGCGTTGAGAG<br>Reverse: TGAAGGAAGATCAACCTCTGC
Bra001598	At3g15540	IAA19	Forward: GGGACGGAGAAGATGATGAA<br>Reverse: TTCCACCGGTAAGAGCAAAC
Bra024536	At1g22770	GI	Forward: GGTGTGAGAGGCACCGTATT<br>Reverse: TGGCAAGTGCATCTGATCTC

.

Supplementary Table S2. The GSL-related genes showing differential expression in GK1 line compared to DH03 wild type

Geneid	arabi_name	arabi_symbol	logFC	logCPM	LR	PValue	FDR	DB_Light	GK_Light
Bra029350.1	AT5G60890	MYB34	*3.484634	12.95044	117.7258	1.99×10^{-27}	2.62E-26	10.32844667	13.75470215
Bra029311.1	AT5G61420	MYB28	1.884898	13.89298	99.10503	2.39×10^{-23}	2.00E-22	12.64614895	14.55008437
Bra012961.1	AT5G61420	MYB28	1.579274	10.40518	33.46055	7.27×10^{-09}	3.52E-08	9.420927498	10.99042762
Bra003726.1	AT1G74090	ST5b	1.524373	9.51849	25.02491	5.66×10^{-07}	1.93E-06	8.545049242	10.09492886
Bra036703.1	AT2G20610	SUR1	1.498236	14.51517	99.7355	1.74×10^{-23}	1.60E-22	13.58095294	15.07827786
Bra000847.1	AT4G03070	AOP1	1.441925	7.369526	8.033005	0.004593	0.010835378	6.116993206	7.754025843
Bra020459.1	AT5G57220	CYP81F2	1.310641	7.281219	5.497721	1.90×10^{-02}	3.81E-02	5.92529697	7.519475298
Bra030696.1	AT1G07640	Dof1.1	0.670766	12.24572	10.46758	0.001215	0.003384911	11.86834446	12.52902904
Bra003645.1	AT1G78370	GSTU20	0.549788	16.80367	10.41354	0.001251	0.003384911	16.49178211	17.04358165
Bra024068.1	AT4G30530	GGP1	0.537439	13.80681	8.329293	0.003901	0.009699992	13.49627278	14.04635109
Bra019332.1	AT4G23100	GSH1/PAD2	0.481821	14.95267	8.254383	0.004065	0.009842478	14.680047	15.17073342
Bra031802.1	AT5G65940	CHY1	0.463419	15.19838	8.537458	3.48×10^{-03}	8.89E-03	14.94149922	15.40819398
Bra016553.1	AT1G18570	MYB51	0.458094	13.91932	5.903977	0.015107	0.031586812	13.65581289	14.120528
Bra021743.1	AT2G31790	UGT74C1	0.453401	14.85442	7.654305	0.005664	0.012708791	14.59901348	15.06233707
Bra021673.1	AT2G30860	GSTF9	0.424034	15.56708	6.770916	0.009266	0.019823921	15.33606178	15.75363254
Bra029966.1	AT3G49680	BCAT-3	0.401492	13.39714	5.31993	2.11×10^{-02}	4.13E-02	13.17434456	13.57945907
Bra039975.1	AT5G65940	CHY1	0.384259	13.61975	5.792153	0.016098	0.03291119	13.41117722	13.79666428
Bra024634.1	AT1G24100	UGT74B1	-0.52908	14.24867	7.908749	0.00492	0.011315147	14.47406304	13.94418136
Bra022815.1	AT2G30860	GSTF9	-0.57987	15.08681	14.38479	1.49×10^{-04}	4.42E-04	15.34491505	14.76057909
Bra016908.1	AT4G13770	CYP83A1	-0.90867	13.81189	32.58498	1.14×10^{-08}	5.00E-08	14.18679215	13.28040224
Bra025668.1	AT1G18590	ST5c	-0.92225	11.34982	17.25352	3.27×10^{-05}	0.000100308	11.70652959	10.78366372
Bra017872.1	AT4G39940	APK2	-1.0374	10.58474	12.87877	0.000332	0.000955451	10.93084583	9.900005834
Bra030246.1	AT2G22330	CYP79B3	-1.05295	9.507773	9.619205	0.001926	0.005061396	9.801246259	8.791671079
Bra011821.1	AT4G39950	CYP79B2	-1.15929	12.81772	27.24066	1.80×10^{-07}	6.61E-07	13.24011855	12.10982785

Bra005641.1	AT2G31790	UGT74C1	-1.1892	13.31133	41.31591	1.30×10^{-10}	7.01E-10	13.76614284	12.58707844
Bra022920.1	AT2G25450	GSL-OH	-1.22643	12.21437	27.37805	1.67×10^{-07}	6.41E-07	12.66072316	11.44702965
Bra015935.1	AT1G74100	ST5a	-1.29528	10.0991	21.10502	4.35×10^{-06}	1.38E-05	10.51741937	9.229288612
Bra032010.1	AT3G03190	GSTF11	-1.47212	11.07868	32.69344	1.08×10^{-08}	4.96E-08	11.57173739	10.11214604
Bra029349.1	AT5G60890	MYB34	-1.55368	11.98437	29.24991	6.36×10^{-08}	2.54E-07	12.52023875	10.91373681
Bra011822.1	AT4G39940	APK2	-1.81463	9.13252	26.28218	2.95×10^{-07}	1.04E-06	9.605687827	7.77778346
Bra015938.1	AT1G74090	ST5b	-2.14184	10.71447	50.47696	1.21×10^{-12}	7.40E-12	11.32003062	9.213568952
Bra018392.1	AT5G65940	CHY1	-2.20326	6.659277	7.289995	0.006934	0.015188688	6.377495624	3.995112345
Bra017871.1	AT4G39950	CYP79B2	-2.4071	9.104987	32.29444	1.32×10^{-08}	5.54E-08	9.61582817	7.187450745
Bra032734.1	AT4G13770	CYP83A1	-3.32778	13.45548	199.1792	3.15×10^{-45}	1.45E-43	14.28026627	10.96981113
Bra026058.1	AT1G16410	CYP79F1	-5.54347	10.34002	113.6079	1.59×10^{-26}	1.83E-25	11.12926523	5.332179498
Bra022448.1	AT3G19710	BCAT-4	-5.6921	8.930608	56.31012	6.19×10^{-14}	4.07E-13	9.487793989	3.771032463
Bra013009.1	AT5G23010	MAM1	-5.93314	10.86604	137.3763	9.98×10^{-32}	1.84E-30	11.68295776	4.839363839
Bra023450.1	AT5G14200	IPMDH1	-6.12114	10.14797	127.0102	1.85×10^{-29}	2.83E-28	10.93599808	4.911044365
Bra034180.1	AT4G03070	AOP1	-6.15328	9.813632	96.54383	8.73×10^{-23}	6.69E-22	10.54025146	3.996516961
Bra013011.1	AT5G23010	MAM1	-6.18621	6.843961	24.92362	5.96×10^{-07}	1.96E-06	7.078421451	1.992907216
Bra029434.1	AT4G12030	BAT5	-6.6243	7.177691	33.67919	6.50×10^{-09}	3.32E-08	7.58819596	1.992907216
Bra018521.1	AT4G03070	AOP1	-6.6879	9.829091	90.94274	1.48×10^{-21}	1.05E-20	10.50631575	3.704850946
Bra029355.1	AT5G23010	MAM1	-6.97451	10.93898	160.3161	9.65×10^{-37}	2.22E-35	11.76643428	4.28677836
Bra001761.1	AT3G19710	BCAT-4	-7.64621	8.020603	42.33018	7.71×10^{-11}	4.43E-10	8.374435818	1.992907216
Bra004744.1	AT2G43100	IPMI SSU2	-8.4375	11.47085	232.8347	1.44×10^{-52}	1.32E-50	12.3536332	3.704850946
Bra000760.1	AT4G12030	BAT5	-8.50065	8.761579	99.80091	1.69×10^{-23}	1.60E-22	9.492734311	1.992907216
Bra000848.1	AT4G03070	AOP1	-10.1839	10.29901	168.956	1.25×10^{-38}	3.84E-37	11.12841507	1.992907216

Color of LogFC values generated by conditional formatting in excel

Supplementary Table S3. The circadian clock genes showing differential expression in GK1 line compared to DH03 wild type

Geneid	arabi_name	arabi_symbol	logFC	logCPM	LR	PValue	FDR	DB_Light	GK_Light
Bra009768.1	AT5G24470	PRR5	*2.740793	10.05995	121.5995	2.82E-28	2.32E-27	8.02147757	10.8010178
Bra038832.1	AT2G18915	LKP2	1.666366	7.075145	5.325339	0.021018	0.041034203	4.12417916	7.0228193
Bra029407.1	AT5G24470	PRR5	1.174672	8.832789	12.82294	3.42E-04	1.08E-03	7.93259927	9.14993585
Bra036517.1	AT5G24470	PRR5	1.127962	10.73359	41.55216	1.15E-10	5.23E-10	9.99470399	11.1475131
Bra028861.1	AT5G02810	PRR7	0.619576	15.5057	61.08057	5.48E-15	3.21E-14	15.1608065	15.7779806
Bra007774.1	AT2G25930	ELF3	0.480213	13.87988	28.69591	8.47E-08	3.47E-07	13.6147001	14.0892657
Bra035933.1	AT5G61380	PRR1	0.377047	11.00215	5.715317	0.016818	0.036290459	10.7193727	11.1486901
Bra030568.1	AT1G04400	CRY2	0.372624	15.29748	21.77358	3.07E-06	1.05E-05	15.0959106	15.4689442
Bra031672.1	AT1G09570	PHYA	0.331351	12.40553	8.673613	0.003229	0.008273043	12.2044842	12.5458375
Bra020017.1	AT1G09530	PIF3	0.324178	13.53956	12.07872	0.00051	0.001493587	13.3638028	13.6873647
Bra002512.1	AT5G60100	PRR3	0.206519	14.2845	5.804735	0.015983	0.036290459	14.1682666	14.3792543
Bra005751.1	AT5G02840	RVE4	-0.27773	13.09359	7.783753	5.27E-03	1.27E-02	13.2159051	12.9345584
Bra005541.1	AT2G32950	COP1	-0.27786	14.62507	11.10272	0.000862	0.002356166	14.7536399	14.4762328
Bra040484.1	AT2G46790	PRR9	-0.39918	15.31905	25.00053	5.73E-07	2.14E-06	15.4996888	15.1033434
Bra004507.1	AT2G46790	PRR9	-0.67153	12.91459	42.12613	8.56E-11	4.39E-10	13.1985003	12.5292769
Bra004503.1	AT2G46830	CCA1	-0.73373	16.32956	90.71035	1.66E-21	1.14E-20	16.6483229	15.9072636
Bra029778.1	AT3G09600	RVE8	-1.20645	14.03794	178.8767	8.52E-41	8.74E-40	14.5050322	13.2953346
Bra033291.1	AT1G01060	LHY	-1.23869	15.43756	234.6164	5.87E-53	8.02E-52	15.9245489	14.6784298
Bra030496.1	AT1G01060	LHY	-1.2517	15.60252	243.2781	7.58E-55	1.55E-53	16.0926443	14.8399026
Bra024536.1	AT1G22770	GI	-1.86509	14.15465	408.7731	6.78E-91	2.78E-89	14.79436	12.9301733
Bra021818.1	AT2G32950	COP1	-3.51823	6.217063	5.377881	0.020394	0.041034203	4.77241845	2.80240314

Color of LogFC values generated by conditional formatting in excel

Supplementary Table S4. The light regulation related genes showing differential expression in GK1 line compared to DH03 wild type

Geneid	arabi_name	arabi_symbol	logFC	logCPM	LR	PValue	FDR	DB_Light	GK_Light
Bra001598.1	AT3G15540	IAA19	*3.296277	8.568363	67.55585	2.05E-16	8.60E-15	5.94325583	9.28739398
Bra029198.1	AT5G63860	UVR8	1.682524	12.06351	249.0334	4.22E-56	3.54E-54	10.9940038	12.6771508
Bra033315.1	AT1G02340	HFR	0.640489	11.6638	34.1994	4.97E-09	1.04E-07	11.3028613	11.9437476
Bra004712.1	AT2G42870	PAR1	0.597764	10.12691	7.359548	0.006671	0.024362762	9.75086867	10.3496405
Bra037742.1	AT2G43010	PIF4	0.585331	12.6883	27.77963	1.36E-07	1.90E-06	12.3617349	12.94438
Bra027232.1	AT3G15540	IAA19	0.518755	10.98869	7.886344	0.004981	0.019018154	10.6817597	11.2000461
Bra008204.1	AT1G75540	BBX21	0.484499	9.810718	7.106985	7.68E-03	2.54E-02	9.51822217	10.0021285
Bra037755.1	AT5G63860	UVR8	0.477489	12.01197	17.17437	3.41E-05	0.000238704	11.7459206	12.2257033
Bra030568.1	AT1G04400	CRY2	0.474956	14.29481	32.07944	1.48E-08	2.49E-07	14.035799	14.5103175
Bra031672.1	AT1G09570	PHYA	0.434929	11.39857	9.386221	0.002186	0.009182143	11.1444336	11.5871578
Bra020017.1	AT1G09530	PIF3	0.425977	12.53514	22.82796	1.77E-06	1.49E-05	12.3037142	12.7287185
Bra024242.1	AT5G63860	UVR8	0.417644	13.58065	26.11833	3.21E-07	3.85E-06	13.352476	13.7730843
Bra016854.1	AT2G42870	PAR1	0.395085	11.64974	5.73863	0.016596	0.04368263	11.4301356	11.809728
Bra003640.1	AT1G78600	BBX22	0.378575	10.78515	7.15698	0.007467	0.025415474	10.5655927	10.9488859
Bra000283.1	AT2G43010	PIF4	0.24525	14.5012	14.49723	0.00014	0.000842197	14.3719055	14.6178131
Bra013286.1	AT4G18130	PHYE	0.225554	12.76924	6.86663	8.78E-03	2.73E-02	12.6480225	12.8722086
Bra037880.1	AT4G08920	CRY1	0.176212	16.57134	7.063573	0.007867	0.025415474	16.4803034	16.6550484
Bra005541.1	AT2G32950	COP1	-0.17626	13.61183	5.84355	0.015634	0.04368263	13.693531	13.5175982
Bra004545.1	AT2G46340	SPA1	-0.20297	15.29013	6.465381	0.011	0.032998815	15.3868668	15.1803181
Bra027259.1	AT3G15354	SPA3	-0.24699	14.20923	13.8692	0.000196	0.001097514	14.3251528	14.0771962
Bra033689.1	AT5G43630	TZP	-0.25659	13.8345	9.631905	0.001912	0.00845421	13.9521134	13.6969161
Bra033689.1	AT5G43630	TZP	-0.25659	13.8345	9.631905	0.001912	0.00845421	13.9521134	13.6969161
Bra008976.1	AT5G11260	HY5	-0.29146	11.69598	5.733846	0.016641	0.04368263	11.8189756	11.5273426
Bra021100.1	AT3G15354	SPA3	-0.32163	12.9187	10.69333	1.08E-03	5.31E-03	13.0645062	12.7361331

Bra001815.1	AT3G21150	BBX32	-0.44413	11.05684	5.774855	0.016257	0.04368263	11.2225499	10.7946246
Bra012972.1	AT5G61270	PIF7	-0.47454	13.66225	23.68801	1.13E-06	1.06E-05	13.873157	13.3969376
Bra001671.1	AT3G17609	HYH	-0.532	13.63196	16.95186	3.83E-05	0.000247734	13.868951	13.3199154
Bra031255.1	AT3G21150	BBX32	-0.69737	9.595364	10.90194	0.000961	0.005043349	9.83421916	9.14278869
Bra013437.1	AT4G19990	FRS1	-0.71947	8.825582	8.217046	4.15E-03	1.66E-02	9.03894304	8.32243917
Bra021258.1	AT3G17609	HYH	-0.79647	11.44275	22.02441	2.69E-06	2.06E-05	11.7626405	10.9320536
Bra022225.1	AT3G17609	HYH	-1.0074	11.01332	57.28809	3.76E-14	1.05E-12	11.3991891	10.3929399
Bra037312.1	AT4G00050	PIF8	-1.14072	9.283742	24.28048	8.33E-07	8.74E-06	9.64713781	8.49199403

Color of LogFC values generated by conditional formatting in excel
